# Changes in Rumen Microbial Community Composition during Adaption to an *In Vitro* System and the Impact of Different Forages

**DOI:** 10.1371/journal.pone.0150115

**Published:** 2016-02-29

**Authors:** Melanie B. Lengowski, Karin H. R. Zuber, Maren Witzig, Jens Möhring, Jeannette Boguhn, Markus Rodehutscord

**Affiliations:** 1 Institut für Nutztierwissenschaften, Universität Hohenheim, Stuttgart-Hohenheim, Baden-Württemberg, Germany; 2 Institut für Kulturpflanzenwissenschaften, Fachgebiet Biostatistik, Universität Hohenheim, Stuttgart-Hohenheim, Baden-Württemberg, Germany; University Paris South, FRANCE

## Abstract

This study examined ruminal microbial community composition alterations during initial adaption to and following incubation in a rumen simulation system (Rusitec) using grass or corn silage as substrates. Samples were collected from fermenter liquids at 0, 2, 4, 12, 24, and 48 h and from feed residues at 0, 24, and 48 h after initiation of incubation (period 1) and on day 13 (period 2). Microbial DNA was extracted and real-time qPCR was used to quantify differences in the abundance of protozoa, methanogens, total bacteria, *Fibrobacter succinogenes*, *Ruminococcus albus*, *Ruminobacter amylophilus*, *Prevotella bryantii*, *Selenomonas ruminantium*, and *Clostridium aminophilum*. We found that forage source and sampling time significantly influenced the ruminal microbial community. The gene copy numbers of most microbial species (except *C*. *aminophilum*) decreased in period 1; however, adaption continued through period 2 for several species. The addition of fresh substrate in period 2 led to increasing copy numbers of all microbial species during the first 2–4 h in the fermenter liquid except protozoa, which showed a postprandial decrease. Corn silage enhanced the growth of *R*. *amylophilus* and *F*. *succinogenes*, and grass silage enhanced *R*. *albus*, *P*. *bryantii*, and *C*. *aminophilum*. No effect of forage source was detected on total bacteria, protozoa, *S*. *ruminantium*, or methanogens or on total gas production, although grass silage enhanced methane production. This study showed that the Rusitec provides a stable system after an adaption phase that should last longer than 48 h, and that the forage source influenced several microbial species.

## Introduction

The rumen hosts a complex microbial community comprised mainly of anaerobic bacteria, methanogens, protozoa, and fungi. These microorganisms break down feed constituents while producing primarily volatile fatty acids, microbial biomass, and gases. The composition of the microbial community in the rumen and the end products of fermentation depend on the diet fed to the animals [1, 2]. For the evaluation of dietary effects on ruminal fermentation, microbial populations and microbial crude protein (CP) synthesis *in vitro* systems are widely used [3–5] to avoid expensive and time-consuming experiments with animals. In addition, *in vitro* systems permit the realization of a large number of treatments in sufficient replication within a relatively short period, along with the testing of higher levels of feed additives that might, in some cases, be potentially toxic to the animals [6]. Furthermore, employing *in vitro* systems allows the establishment of well-controlled environmental testing conditions, avoiding the variability inherent when utilizing individual animals [7].

One commonly used *in vitro* system is the semi-continuous rumen simulation technique (Rusitec) developed by Czerkawski and Breckenridge [7]. These authors reported similar types and quantities of fermentation products *in vitro* as those generated by the rumen of animals used as rumen content donors. Recently Martinez et al. [8, 9] compared certain characteristics of fermentation and microbial community composition in a Rusitec system presented with different concentrate to forage ratios and types of forages to those found in sheep in order to investigate how closely fermenters can mimic the dietary differences found *in vivo*. Differences between fermenters and animals were detected but the authors also reported that the Rusitec system simulated the *in vivo* fermentation more closely when high-forage rather than high-concentrate diets were used. Although Rusitec fermenters did not maintain protozoa numbers at levels found *in vivo*, sheep and fermenters showed similar total numbers of bacteria with high-forage diets. Different conditions between fermenters and animals may cause a preferential selection of certain bacterial strains *in vitro* [9]. Ziemer et al. [10] examined the adaption of the ruminal microbial community to a dual-flow continuous culture system during the first 240 h of incubation and identified a divergent microbial community at the end of the adaption phase compared to that in the inoculum. However, despite the identified changes in the microbial community composition, the model system used in this study supported a functional community structure similar to that found in the rumen. In contrast, studies on adaption of the microbial community in a Rusitec system have been restricted to the examination of fermentation characteristics [11]. Furthermore, studies on the diurnal changes of the ruminal microbial community composition in Rusitec systems are rare [12, 13]. Hence, the first objective of the present study was to investigate the changes of different microbial groups during adaption to the Rusitec system within the first two days of incubation and to study the diurnal changes of the microbial populations after adaptation using two different forages.

Forages in ruminant rations account for at least 40% of the ration and different forage sources have been shown to affect the microbial community composition differently both *in vivo* [14] and *in vitro* [9]. In Europe and North America grass silage (GS) and corn silage (CS) are the most important silages used in dairy cows and fattening cattle feeding. Owing to their different nutrient compositions, these silages have diverse effects on ruminal fermentation and the microbial community *in vivo* [15, 16] and *in vitro* [17, 18]. However, in most former studies the silages were combined with concentrates. Therefore, the second objective of this study was to investigate the effects on the ruminal microbial community and fermentation when incubating only GS or CS without concentrates.

## Materials and Methods

### Ethic statement

The cows used as donor animals for the inoculum in this study were housed at the Agricultural Experiment Station of Hohenheim University, location Meiereihof in Stuttgart (Germany), in strict accordance with the German Animal Welfare legislation. All procedures regarding animal handling and treatments within this study were approved by the Ethical Commission of Animal Welfare of the Provinical Government of Baden- Württemberg, Germany.

### *In vitro* experiment

The *in vitro* experiment was carried out using a semi-continuous Rusitec system and followed the procedures described by Boguhn et al. [19]. Three lactating cows (Jersey; 500 ± 61.9 kg of body weight and milk production of 24.3 ± 2.89 l day^-1^) fitted with permanent rumen cannulas were used as donor animals for the inoculum. Two of the three donor cows were in mid-lactation and one in early-lactation two of them being in third and one in fourth lactation. The cows were offered hay and a total mixed ration containing CS and GS for *ad libitum* consumption. The inoculum was obtained from the solid and liquid phases of the rumen before the provision of new feed. Rumen contents from the three cows were mixed and filtered through two layers of linen cloth. Two Rusitec systems each comprising six fermenters in a water bath (39°C) were used in this study. The fermenters were filled with 800 ml of a 1:1 mixture of rumen liquid and artificial saliva [20] containing 0.7 mmol l^−1^ NH_4_^+^ from NH_4_Cl (enriched with 10.39% ^15^N; Campro Scientific GmbH, Berlin, Germany).

Silages with nutrient specifications as shown in Table 1 were oven-dried (24 h at 65°C) and ground through a 1 mm sieve; 15 g ground silage was used to fill individual nylon bags (pore size = 100 μm, Fa. Linker Industrie-Technik GmbH, Kassel, Germany). For each silage one fermenter was used and five experimental replicates (*n* = 5) were carried out. The experimental replicates were started on five consecutive days to consider the daily variations in microbial communities that naturally occur in the rumen of the donor animals. At the beginning of each experimental run, fermenter was filled with only one bag of the corresponding silage whereas the second contained pooled rumen solids (60 ± 5 g). After 24 h, the latter was replaced by a second feedbag. On the following days, feedbags were replaced at 24-h intervals so that each bag was incubated for 48 h in total. Within each experimental run, an additional fermenter served as the blank control containing only one bag filled with pooled rumen solids for 24 h; this fermenter was run for the initial 48 h of incubation. Each experimental run lasted for 13 days. During the experiment, artificial saliva was continuously infused at a rate of approximately 590 ml day^−1^. Vertical movement of the feed containers inside the fermenters was achieved by an electric motor with 10 to 12 strokes min^−1^. The effluent was collected in 1 l bottles standing inside an ice-cold water bath. The gas produced was collected in 10 l bags (Linde PLASTIGAS^®^-bags, Linde AG, Pullach, Germany) for quantification of gas production and methane concentrations as described previously [21].

**Table 1:** Chemical composition of the silages used for incubation.

	CS^a^	GS^b^
**Dry matter (DM), %**	93.7	90.4
**Crude ash, % DM**	5.0	8.0
**Crude protein, % DM**	8.1	17.1
**NDF**^c^**, % DM**	39.8	41.3
**ADF**^d^**, % DM**	25.1	26.4
**ADL**^e^**, % DM**	2.0	1.5
**Starch, % DM**	31.6	-

^a^Corn silage,

^b^Grass silage,

^c^Neutral detergent fiber without residual ash after α amylase pretreatment,

^d^Acid detergent fiber,

^e^Acid detergent lignin.

### Sampling

Samples were taken within two time periods during each Rusitec run. In period 1, fermenter liquid (40 ml) was collected from the fermenters at 0, 2, 4, 12, 24, and 48 h after starting the incubation. A 30 ml subsample was stored at −20°C for determination of the ammonium concentration and 1 ml aliquots were stored at −80°C for microbial DNA extraction. Samples of rumen solids were obtained at the beginning of each Rusitec run and from feed residues in the bags after 24 and 48 h of incubation, and were stored at −80°C. Starting on day 7 of incubation the total amount of effluent, gas production, methane, and feed residues were quantified on a daily basis until day 13 (period 2). A 70 ml sample of the effluent from each fermenter was collected each day and later pooled over days 7 to 13. For removal of feed particles and microbes, the effluent was centrifuged at 27 000 × *g* at 4°C for 15 min using a Sorvall RC-5B Refrigerated Superspeed Centrifuge (GMI, Ramsey, Minnesota, USA). The particle-free fraction was stored at −20°C for subsequent analysis of short-chain fatty acids (SCFA), ammonia-N, and ^15^N enrichment. The feed residues obtained from the nylon bags were dried for 24 h at 65°C and pooled over days 7 to 12 for the analysis of nutrient fractions according to the official methods in Germany [22]. To determine the microbial CP synthesis, 30 ml of fermenter liquids were collected daily from each fermenter and pooled over days 7 to 13 to obtain liquid-associated microbes (LAM) by differential centrifugation according to Brandt and Rohr [23] with modifications as described by Wischer et al. [24]. After centrifugation the microbial pellets were frozen at −20°C until analysis for ^15^N enrichment. Solid-associated microbes were separated from feed residues on day 13 of incubation as described by Boguhn et al. [25]. Microbial pellets were stored at −20°C for the subsequent analysis of ^15^N enrichment. Samples for microbial community analysis in period 2 were taken within the last 24 h of incubation. Fermenter liquid was collected at 0, 2, 4, 12, and 24 h after changing the feedbag on day 12. Samples from feed residues were collected at the end of each Rusitec run after 24 and 48 h of incubation. Samples for DNA extractions were stored at −80°C.

### Chemical analyses

Feed residues from the bags were ground to pass through a sieve of 0.5 mm pore size and analyzed for dry matter by oven-drying for 4 h at 103°C (method 3.1) and crude ash by incineration at 550°C for 4 h (method 8.1). To determine CP, the nitrogen concentration was analyzed by the Kjeldahl method comprising acid digestion of the samples with sulfuric acid, steam distillation and determination of the ammonium formed by suitable titration technique. The resulting nitrogen concentration was multiplied by a 6.25 to gain the concentration of CP (method 4.1.1). The samples were analyzed for neutral detergent fiber by boiling for 1 h in a solution of disodium tetraborate, detergents and a thermally stable amylase (method 6.5.1). The acid detergent fiber was determined by boiling the samples for 1 h in sulfuric acid detergent solution (method 6.5.2). Starch (for CS only) was analyzed using a polarimetric approach after heating the samples in diluted hydrochloric acid (method 7.2.1). Methods are described in detail previously [22]. Samples of particle-free effluent and fermenter liquid were analyzed for ammonia concentration by steam distillation followed by end-point titration [24]. Concentrations of SCFA in the particle-free effluent were measured by gas chromatography as described by Geissler et al. [26] using 2-methylvaleric acid as an internal standard. Samples of silages, feed residues, ^15^NH_4_Cl, freeze-dried microbial pellets, and particle-free effluent were ground finely and analyzed for ^15^N and N (only microbial pellets) using an elemental analyzer (EA 1108; Carlo Erba Instruments, Biberach, Germany) combined with an isotope mass spectrometer (MS Finnigan MAT; Thermoquest Italia S.p.A., Milan, Italy). The microbial protein (microbial N multiplied by 6.25) from LAM was calculated as the difference between the input and output of ^15^N divided by the ^15^N concentration in LAM. Microbial protein originating from solid-associated microbes was calculated according to Hildebrand et al. [5]. Calculations of the degradation of nutrient fractions as well as for the efficiency of microbial CP synthesis were performed as described in detail elsewhere [19].

### Real-time quantitative (q)PCR

For quantification of the different microorganisms by real time qPCR, microbial DNA was extracted using the repeated bead-beating method as described by Yu and Morrison [27] with the following modifications: for cell lysis in fermenter liquids, 0.15 g of 0.1 mm and 0.05 g of 0.5 mm sterile zirconia beads were used whereas for cell lysis in feed residues, only 0.05 g of 0.05 mm sterile beads were used. Samples were homogenized using a FastPrep Instrument (MP Biomedicals, Eschwege, Germany) for 40 s at step 4. All centrifugation steps were carried out at room temperature at 16 000 × *g* using a Heraeus Pico 17 centrifuge (Thermo Scientific, Braunschweig, Germany). DNA extracts were stored at −20°C. The integrity of the isolated DNA was checked by agarose gel electrophoresis. The purity of the DNA extracts was assessed spectrophotometrically using a NanoDrop-1000 (NanoDrop Technologies, Inc., Wilmington, DE, USA). DNA extracts showing a relatively low OD_260_/OD_230_ ratio were additionally purified by ethanol precipitation according to Popova et al. [28]. After ethanol precipitation the ratios of OD_260_/OD_280_ and OD_260_/OD_230_ were on average (SD) 1.92 (0.14) and 1.70 (0.35), respectively. The DNA concentration in the extracts was measured fluorometrically using a Qubit^®^ 2.0 Fluorometer and the Qubit^™^ dsDNA BR Assay Kit (Invitrogen, Ltd., Paisley, UK) according to the manufacturer’s protocol.

Conventional PCR was used to generate sample-derived DNA standards for each real-time qPCR assay. For this purpose, a composite DNA sample was prepared by pooling an equal amount of all DNA extracts. The primer sets that were used for the amplification of different species are listed in Table 2. PCR was performed with the iQ^™^5 Multicolor Real-Time PCR Detection System (Bio-Rad, München, Germany) in a total volume of 25 μl containing 5 μl of 5× PCR-Mastermix (Bio & Sell, Feucht/Nürnberg, Germany), 14 ng of template DNA, and primer concentrations ranging from 300–900 nM. The amplification conditions were as follows: initial denaturation at 94°C for 5 min, 30 cycles of denaturation at 94°C (15–30 s), annealing at 55–61°C (30–60 s), and elongation at 72°C (10–120 s), with a terminal elongation step at 72°C for 5 min. The PCR products were separated by agarose gel electrophoresis to confirm the expected fragment length. Amplicons were purified using the MinElute PCR Purification Kit (Qiagen, Hilden, Germany) according to the manufacturer’s protocol and were quantified fluorometrically as described for the DNA extracts. Gene copy numbers were calculated according to Lee et al. [29]. A tenfold serial dilution series of each PCR product with 5–6 degrees of dilution was used for generating standard curves.

**Table 2:** Target organisms, target genes, optimized annealing temperatures (T_a_), and primer concentrations used in real-time quantitative PCR.

Target organism	Target gene	T_a_ (°C)	Primer conc. (nM)	Reference
**Protozoa**	18S rRNA	55	600	[30]
**Methanogens**	mcrA	60	900	[31]
**Domain bacteria**	16S rRNA	50	500	[32]
***Fibrobacter succinogenes***	16S rRNA	58	200	[32]
***Ruminococcus albus***	16S rRNA	55	500	[33]
***Ruminobacter amylophilus***	16S rRNA	60	500	[32]
***Prevotella bryantii***	16S rRNA	61	500	[32]
***Selenomonas ruminantium***	16S rRNA	59	500	[32]
***Clostridium aminophilum***	16S rRNA	56	400	[14, 34]

Quantification of the gene copy numbers in each sample was also performed on the iQ^™^5 thermal cycler. Real-time qPCR assays were optimized for MgCl_2_ and primer concentrations as well as for annealing temperature. Reactions were carried out in a total volume of 20 μl in Framestar 96 well PCR-plates (Bio & Sell). The reaction mixtures contained 4 μl of a 5× *my*-Budget EvaGreenQPCR Mix II (Bio & Sell), 2.5 mM MgCl_2_, 14 ng of template DNA, and primer concentrations as given in Table 2. The amplification for each sample was performed in duplicate and with the following conditions: initial denaturation at 95°C for 15 min, 35–45 cycles of denaturation at 95°C (15–35 s), annealing at 55–61°C (30–60 s), and elongation at 72°C (20–90 s), followed by a terminal elongation step at 72°C for 5 min. Standards were run in triplicate. On every plate, two standard curves were generated, one using PCR products for absolute quantification and one to determine the PCR efficiency in samples using a fivefold serial dilution series with 5 degrees of dilution from the pooled DNA sample. For each experimental run, one plate was run for the fermenter liquids and one for the feed residues. The specificity of amplification was determined by melting curve analysis. To determine the quantification cycle (C_q_), the background subtracted fluorescence data obtained from real-time qPCR were imported to the LinRegPCR quantitative PCR data analysis program (Version 2013.0; Ruijter et al., Department of Anatomy, Embryology & Physiology, Academic Center, Amsterdam, the Netherlands). Differences in C_q_ > 0.5 between the two sample replicates led to exclusion of the sample from further data analysis. Absolute gene copy numbers in the samples were calculated by using the respective standard curves.

### Statistical analysis

Data for absolute gene copy numbers and for ammonia-N were analyzed using a mixed models approach (procedure PROC MIXED of the software package SAS; Version 9.3) considering the two treatment factors (ration and time) and including block effects according to the used design in Rusitec phase and the molecular characterization (qPCR) phase. The ration factor was split into sample classes (C), and forage sources (FS) within silage. The former separates inoculum and blank from silage, the latter distinguish between CS and GS. The time factor was split into period (P) and sampling time (ST) as observations were taken at six sampling times during two periods. An overview of the coding of variables used in the model is given in Table 3. The model in the syntax of Patterson [35] can be represented by:

C + FS⋅C + P + ST⋅P + FS⋅C⋅P + FS⋅C⋅P⋅ST + R + WB : B⋅R + Place⋅WB + F⋅Place⋅WB + P⋅F⋅Place⋅WB

where C, FS, P, and ST denote the treatment factors of sample class, forage source, period, and sampling time, respectively. WB, Place, F, P, R, and B denote the block factors of water bath, place within the water bath, fermenter, period, replicate, and block, respectively. R and B are block effects of the laboratory phase. Fixed effects are presented before the colon, and random effects are given after. Interactions are denoted by a dot between the corresponding main effects. Water bath and replicate effects are assumed to be random, but are taken as fixed because of the low number of values. As the ration inoculum is not included in the Rusitec run, no effects from block factors arising from the Rusitec phase were fitted. A dummy variable was used to eliminate these effects, but is dropped from the model description to simplify the presentation. We accounted for temporal correlations due to repeated measurements from the same fermenter by fitting either a constant covariance over time or an autoregressive model for temporal effects if the latter increased the model fit. Heterogeneous error variance for the sample classes was fitted using independent or autoregressive [36] error structures. The data were logarithmic (concentration of ammonia-N; gene copy numbers in feed residues: Total bacteria, *Ruminococcus albus*, *Fibrobacter succinogenes*, *Prevotella bryantii*; gene copy numbers in fermenter liquids: *P*. *bryantii*, *Ruminobacter amylophilus*) or square root (remaining data on gene copy numbers) transformed to reach normality distributed error with homogeneous variances within a sample class. A multiple t-test for treatment comparisons was used only when the F-test was significant.

**Table 3:** Overview of the coding of variables used in the statistical model^a^.

Ration	Sample class	Forage source	Water bath	Sampling time	Fermenter	Place	d1
**Inoculum**	0	Inoculum	0	0	0	0	0
**Blank**	1	Blank	1 or 2	1 to 6	1 to 15	1 to 12	1
**Silage**	2	CS or GS^b^	1 or 2	1 to 6	1 to 15	1 to 12	1

^a^If more than one value is given, the variable can take any of the given values,

^b^CS: corn silage; GS: grass silage.

Fermentation data were analyzed by a mixed model incorporating silage, replicate, and water bath as fixed effects and fermenter as a random effect using the procedure PROC MIXED of the software package SAS (Version 9.3). Within the third experimental run, the collected total gas amount was much less compared to that obtained from the other runs; thus, the data for gas and methane concentration were omitted from the statistical analysis.

## Results

**Fig 1:**
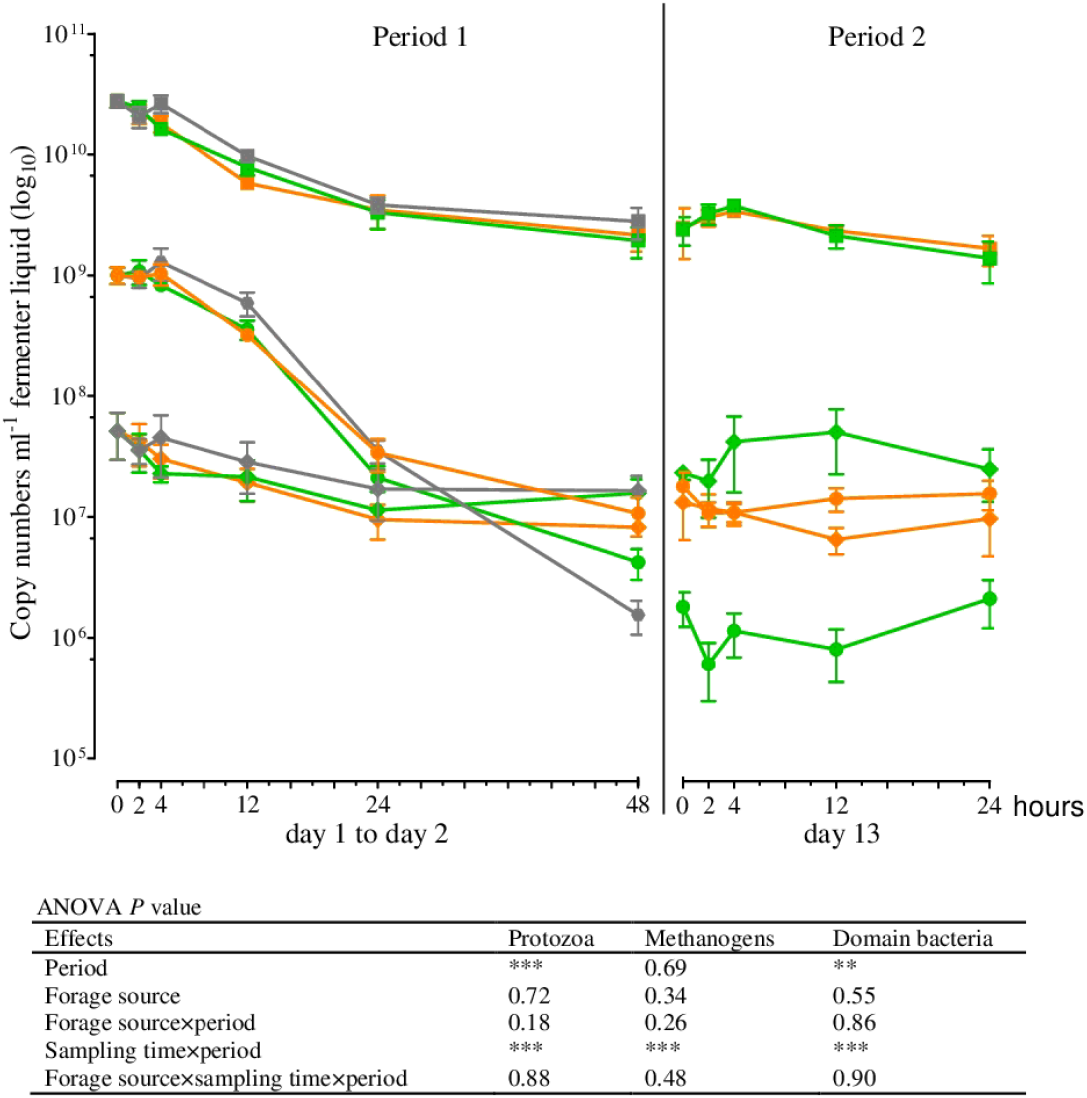
18S rRNA, *mcrA*, and 16S rRNA gene copy numbers in the fermenter liquid for protozoa (●), methanogens (♦), and total bacteria (■) at different times of incubation (Mean, SEM; green: grass silage; orange: corn silage; gray: blank; *n* ≥2; **P ≤ 0.01; ***P ≤ 0.001).

**Fig 2:**
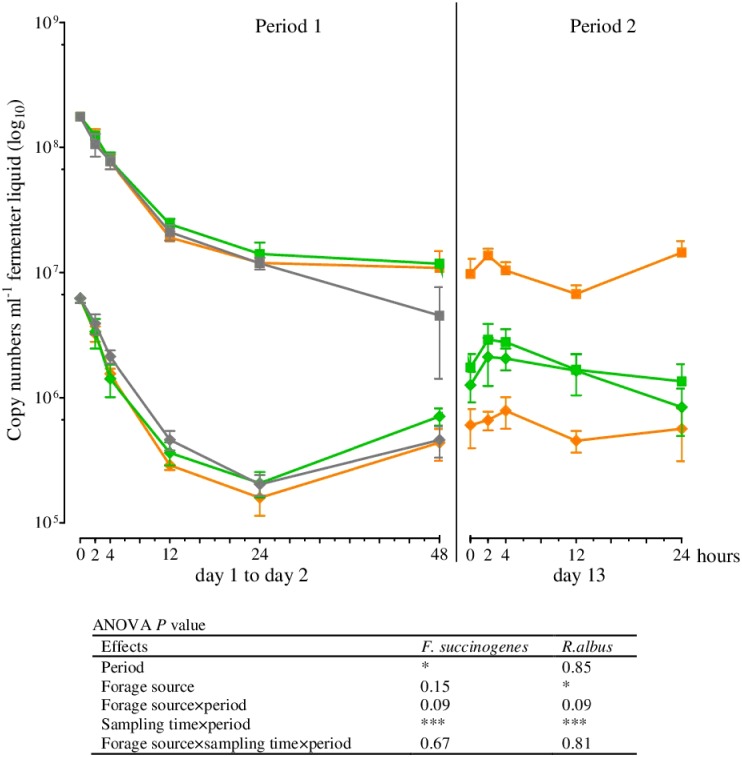
16S rRNA gene copy numbers in the fermenter liquid for *Fibrobacter succinogenes* (■) and *Ruminococcus albus* (♦) at different times of incubation (Mean, SEM; green: grass silage; orange: corn silage; gray: blank; *n* ≥ 4; *P ≤ 0.05; ***P ≤ 0.001).

**Fig 3:**
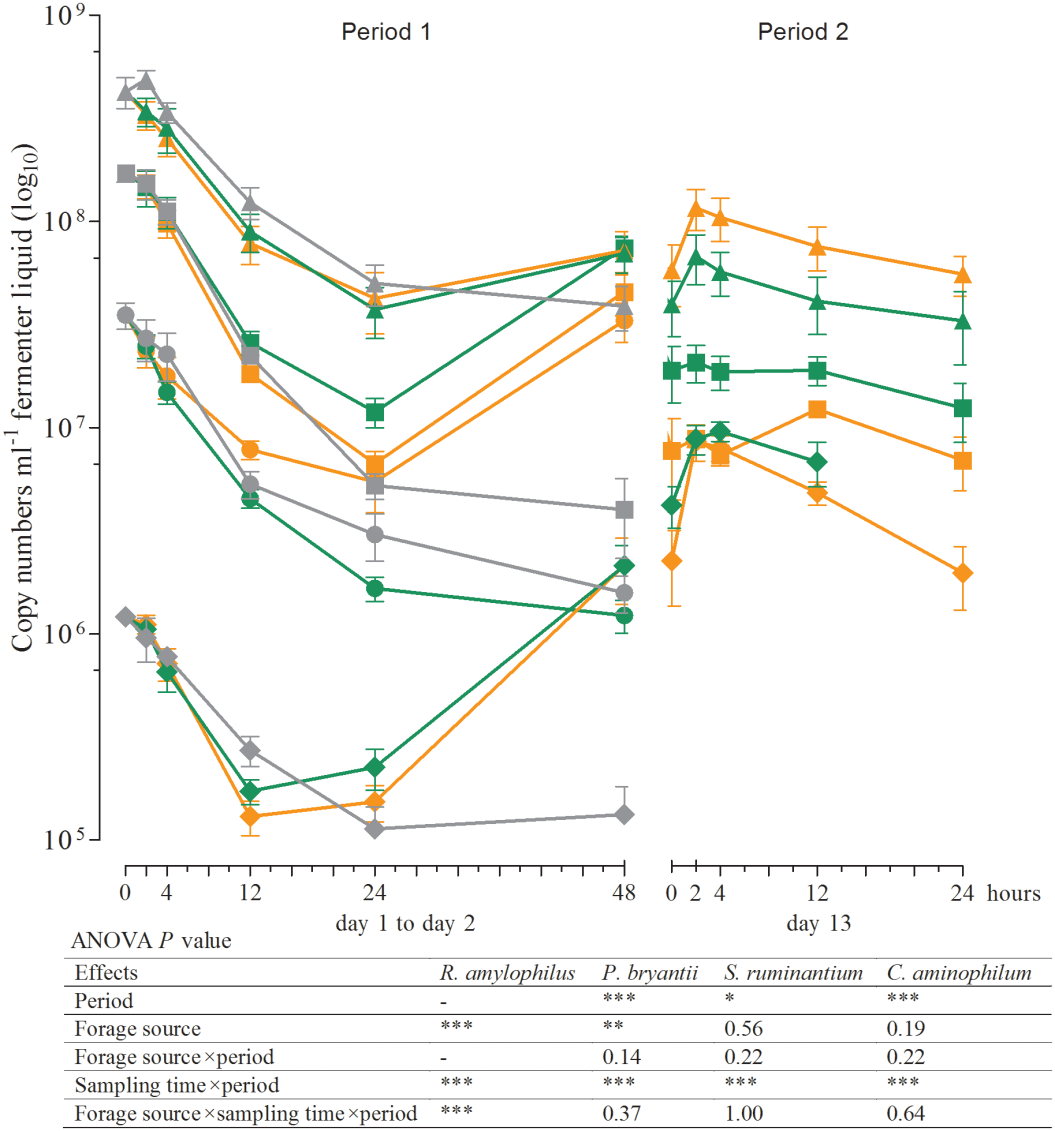
16S rRNA gene copy numbers in the fermenter liquid for *Ruminobacter amylophilus* (●), *Prevotella bryantii* (■), *Selenomonas ruminantium* (▲), and *Clostridium aminophilum* (♦) at different times of incubation (Mean, SEM; green: grass silage; orange: corn silage; gray: blank; *n* ≥ 3; *P ≤ 0.05; **P ≤ 0.01; ***P ≤ 0.001). *R*. *amylophilus* could not be detected in period 2. Owing to insufficient template DNA for amplification, no data are available on the 24 h sampling time in period 2 for *C*. *aminophilum* and GS.

**Fig 4:**
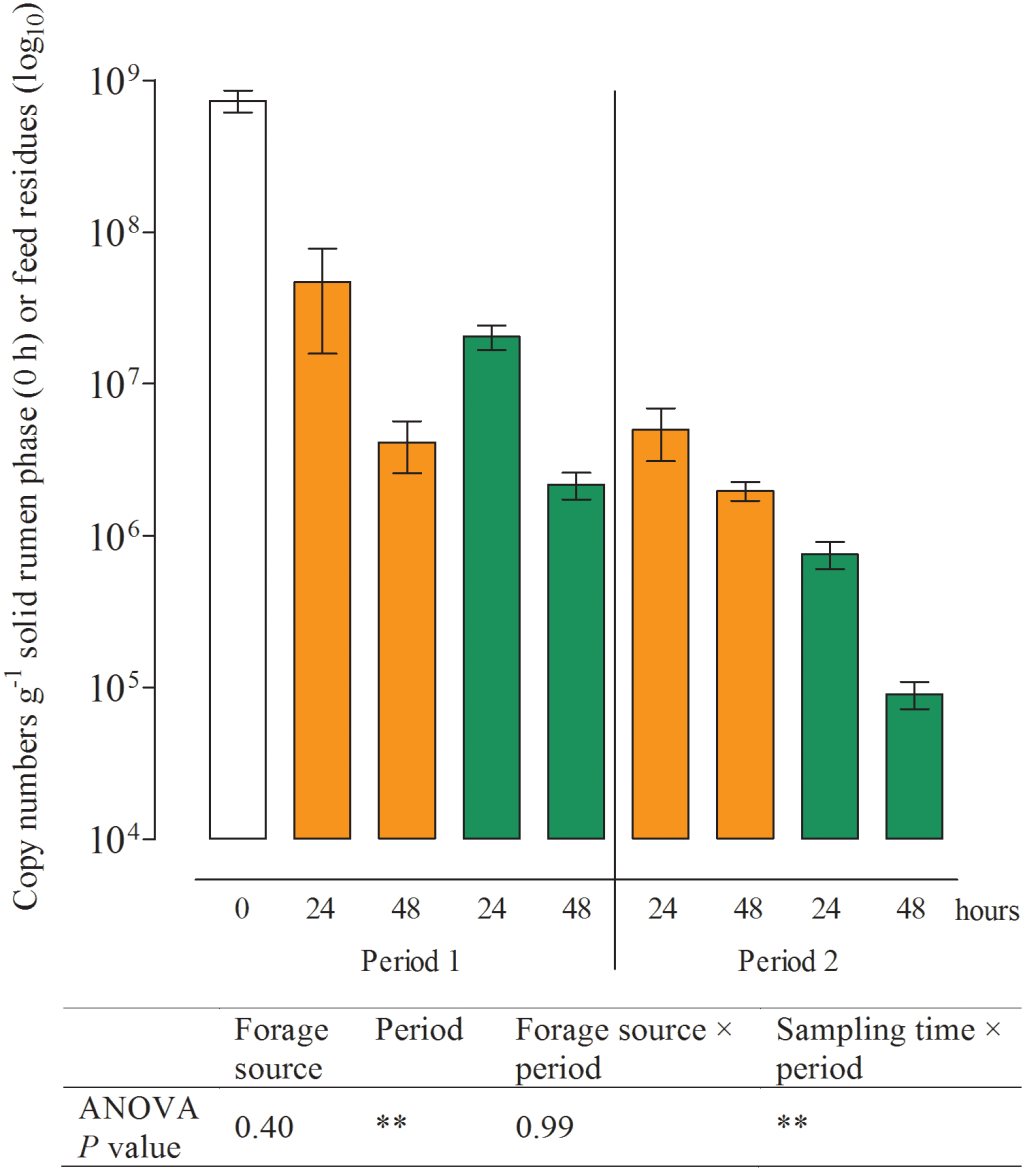
18S rRNA gene copy numbers determined in the solid rumen phase (0 h; white) and feed residues for protozoa at different times of incubation (Mean, SEM; green: grass silage; orange: corn silage; *n* = 5; **P ≤ 0.01).

**Fig 5:**
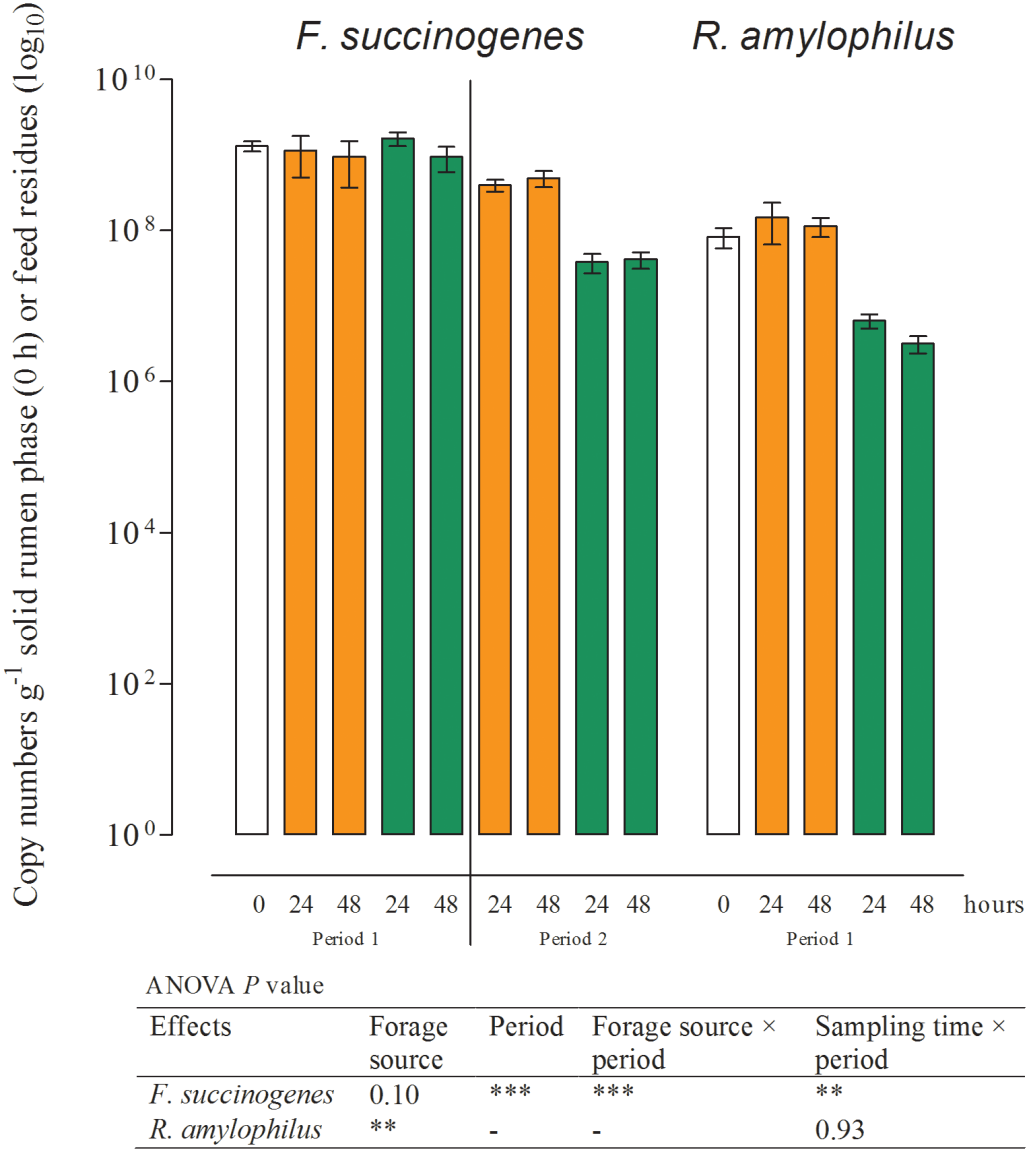
16S rRNA gene copy numbers determined in the solid rumen phase (0 h; white) and feed residues for *Fibrobacter succinogenes* and *Ruminobacter amylophilus* at different times of incubation (Mean, SEM; green: grass silage; orange: corn silage; *n* ≥ 3; **P ≤ 0.01; ***P ≤ 0.001). *R*. *amylophilus* could not be detected in period 2 owing to insufficient template DNA for amplification.

**Fig 6:**
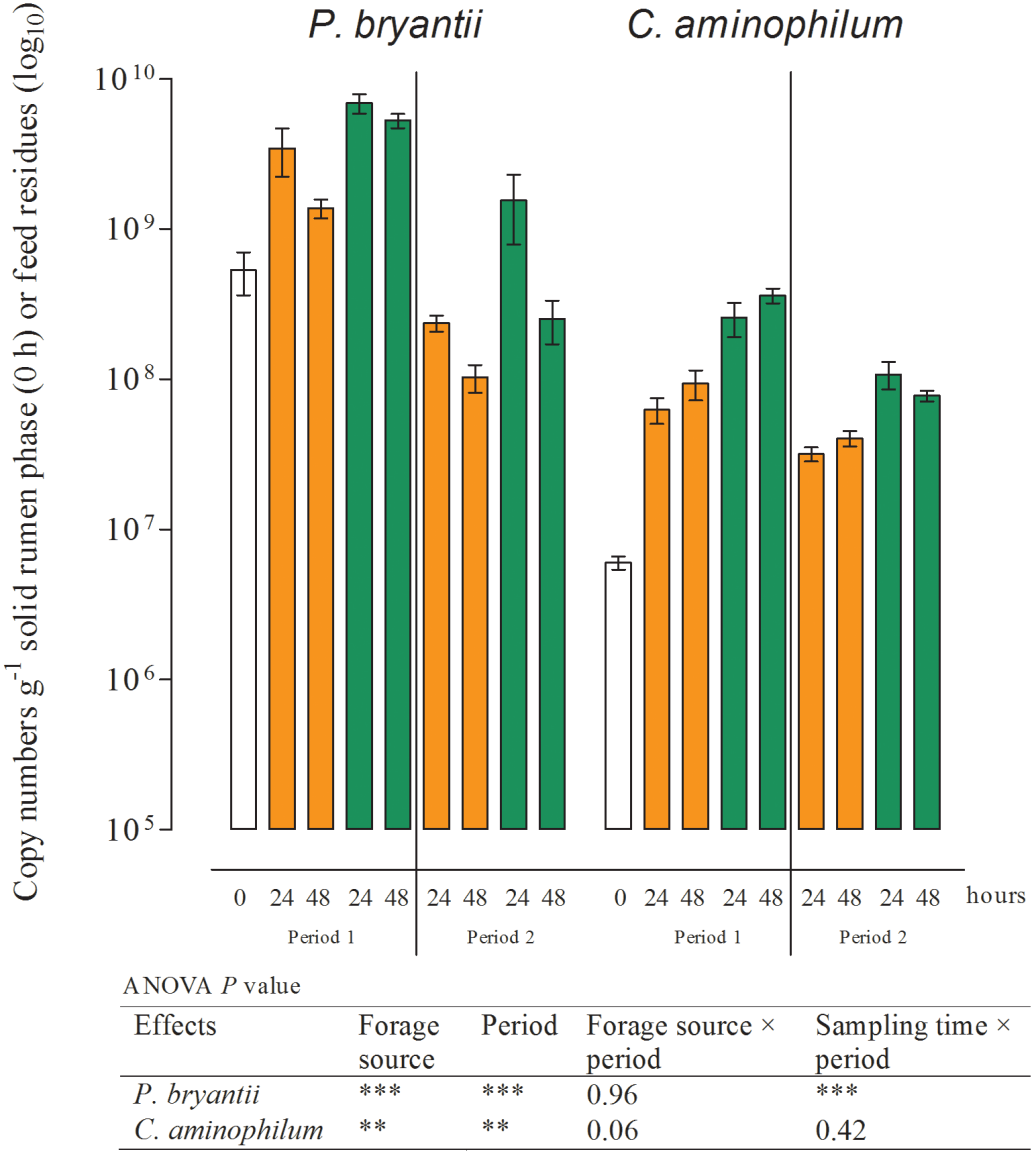
16S rRNA gene copy numbers determined in the solid rumen phase (0 h; white) and feed residues for *Prevotella bryantii* and *Clostridium aminophilum* at different times of incubation (Mean, SEM; green: grass silage; orange: corn silage; *n* ≥ 4; **P ≤ 0.01; ***P ≤ 0.001).

The results of real-time qPCR for the fermenter liquids are shown in Figs 1–3 and those of the solid rumen phase and feed residues in Figs 4–6 and Table 4. They are expressed as 18S rRNA, *mcrA*, and 16S rRNA gene copy numbers ml^−1^ fermenter liquid or g^−1^ solid rumen phase and feed residues, respectively.

**Table 4:** McrA and 16S rRNA gene copy numbers of methanogens, total bacteria, *Ruminococcus albus*, and *Selenomonas ruminantium*.

		Period 1			Period 2		*p*- value			
Microbial group	Forage source	0 h	24 h	48 h	24 h	48 h	Forage source	Period	Forage source× period	Sampling time× period
**Methanogens**	CS^a^	0.96 (0.20)	0.43 (0.24)	2.67 (1.74)	0.15 (0.06)	0.36 (0.22)	n.s.	n.s.	n.s.	n.s.
	GS^b^		0.40 (0.12)	0.75 (0.27)	0.12 (0.05)	0.06 (0.02)				
**Total bacteria**	CS	562 (166)	200 (159)	256 (72.4)	362 (158)	289 (122)	n.s.	n.s.	n.s.	n.s.
	GS		315 (133)	326 (89.2)	314 (169)	247 (62.7)				
***R*. *albus***	CS	0.75 (0.41)	0.26 (0.36)	0.24 (0.24)	0.39 (0.21)	0.25 (0.22)	n.s.	n.s.	n.s.	n.s.
	GS		0.73 (0.66)	0.85 (0.59)	1.00 (0.98)	0.65 (0.48)				
***S*. *ruminantium***	CS	25.1 (2.37)	54.2 (25.1)	70.5 (37.0)	67.9 (12.2)	39.3 (18.2)	n.s.	n.s.	n.s.	n.s.
	GS		89.1 (63.1)	43.3 (16.3)	18.8 (9.07)	12.2 (3.27)				

Data were detected by real-time quantitative PCR and expressed in copy number × 10^8^ g^−1^; mean (SD); *n* ≥4. Gene copy numbers were determined in the solid rumen phase (0 h) and feed residues after 24 and 48 h of incubation.

^a^Corn silage,

^b^Grass silage.

### Effects of period and sampling time on the microbial populations in fermenter liquids

Within the first hours of incubation (period 1), the copy numbers of the 18S rRNA, *mcrA*, and 16S rRNA genes of all microbial species examined decreased irrespective of the incubated silage. The lowest numbers for the methanogens and for *R*. *albus*, *F*. *succinogenes*, *Selenomonas ruminantium*, and *P*. *bryantii* were seen after 24 h of incubation, whereas the minimum for *Clostridium aminophilum* was observed after 12 h and for protozoa and total bacteria after 48 h (Figs 1–3). No significant differences in the gene copy numbers of total bacteria, protozoa, methanogens, and *F*. *succinogenes* were observed between 24 and 48 h of incubation (Figs 1 and 2) but significantly higher abundances of *R*. *albus*, *S*. *ruminantium*, *P*. *bryantii*, and *C*. *aminophilum* (Figs 2 and 3) were observed after 48 h. The number of *R*. *amylophilus* decreased until 48 h for GS, while the minimum was found at 24 h for CS and after 48 h, the numbers returned to the level of the initial inoculum (Fig 3). No significant differences were observed between the silages and the blank control for the methanogens, protozoa, total bacteria, *R*. *albus*, and *S*. *ruminantium* (Figs 1–3). The numbers of *F*. *succinogenes*, *P*. *bryantii*, and *C*. *aminophilum* were significantly lower after 48 h in the blank controls compared either silage (Figs 2 and 3), whereas the numbers of *R*. *amylophilus* in the controls showed no significant difference compared to GS, and lower numbers were found after 48 h of incubation compared to CS (Fig 3).

At the first sampling in period 2 (0 h) the gene copy numbers of the protozoa, methanogens, total bacteria, *R*. *albus*, *S*. *ruminantium*, and *C*. *aminophilum* were at a similar level as those observed after 48 h of incubation in period 1 (Figs 1–3). In contrast, the numbers of *F*. *succinogenes* and *P*. *bryantii* were significantly lower in period 2 than in period 1 (Figs 2 and 3). No data was obtained for *R*. *amylophilus* in period 2 (Fig 3) as only nonspecific products were detected.

After the addition of a fresh feedbag at the beginning of period 2, increasing gene copy numbers in the fermenter liquids for all of the prokaryotes investigated were observed. Irrespective of the incubated silage, the numbers of *F*. *succinogenes* (Fig 2), *S*. *ruminantium*, and *C*. *aminophilum* (Fig 3) increased significantly between 0 and 2 h after feeding, whereas the gene copy numbers of the methanogens, total bacteria (Fig 1), *R*. *albus* (Fig 2), and *P*. *bryantii* (Fig 3) did not differ between sampling times. The protozoa numbers were lowest at 2 h after feedbag substitution (Fig 1); thereafter, the numbers increased up to 24 h after feeding to the levels found at the beginning of period 2. No data was obtained for *C*. *aminophilum* plus GS for the 24 h post-feeding sampling time, as insufficient template DNA was available for amplification (Fig 3). Irrespective of the silage used for incubation, at 24 h the numbers of the remaining microbial species examined were at the same level as at the beginning of period 2 (Figs 1–3).

### Effects of period and sampling time on the microbial populations in feed residues

No effect of period or sampling time was observed for the numbers of particle-associated methanogens, total bacteria, *S*. *ruminantium*, *R*. *albus* (Table 4), and *R*. *amylophilus* (Fig 5) in the feed residues. The number of particle-associated protozoa significantly decreased within the first 48 h of incubation (Fig 4). Similar results were found for *F*. *succinogenes* (Fig 5). Copy numbers for *P*. *bryantii* in period 1 were the lowest in the solid phase of the rumen (0 h) and highest in the feed residues at 24 h after the initiation of incubation (Fig 6). In period 2, the numbers of *P*. *bryantii* were significantly lower than those in period 1, but a decrease between the 24 and 48 h sampling times was also observed. The numbers of *C*. *aminophilum* in the solid rumen phase did not significantly differ between sampling times, but a significantly lower number of this species was observed in period 2 (Fig 6).

### Effects of forage source on the microbial populations

The numbers of protozoa, methanogens, total bacteria, and *S*. *ruminantium* were not affected by the forage source in either the fermenter liquids or the feed residues (Figs 1 and 3; Table 4). Incubation of CS resulted in higher period 2 gene copy numbers of *F*. *succinogenes* in both fermenter liquids as well as feed residues (Figs 2 and 5). In feed residues, higher numbers were found for *R*. *amylophilus* in period 1 during incubation of CS. In the fermenter liquid (Fig 3), an interaction between forage source and sampling time was detected for *R*. *amylophilus* gene copy numbers, wherein higher numbers were found for CS after 24 and 48 h of incubation. In contrast, the numbers of *R*. *albus* (Fig 2, Table 4) and *P*. *bryantii* (Figs 3 and 6) in both sites were increased by GS incubation.

### Effects of forage source on fermentation characteristics

**Fig 7:**
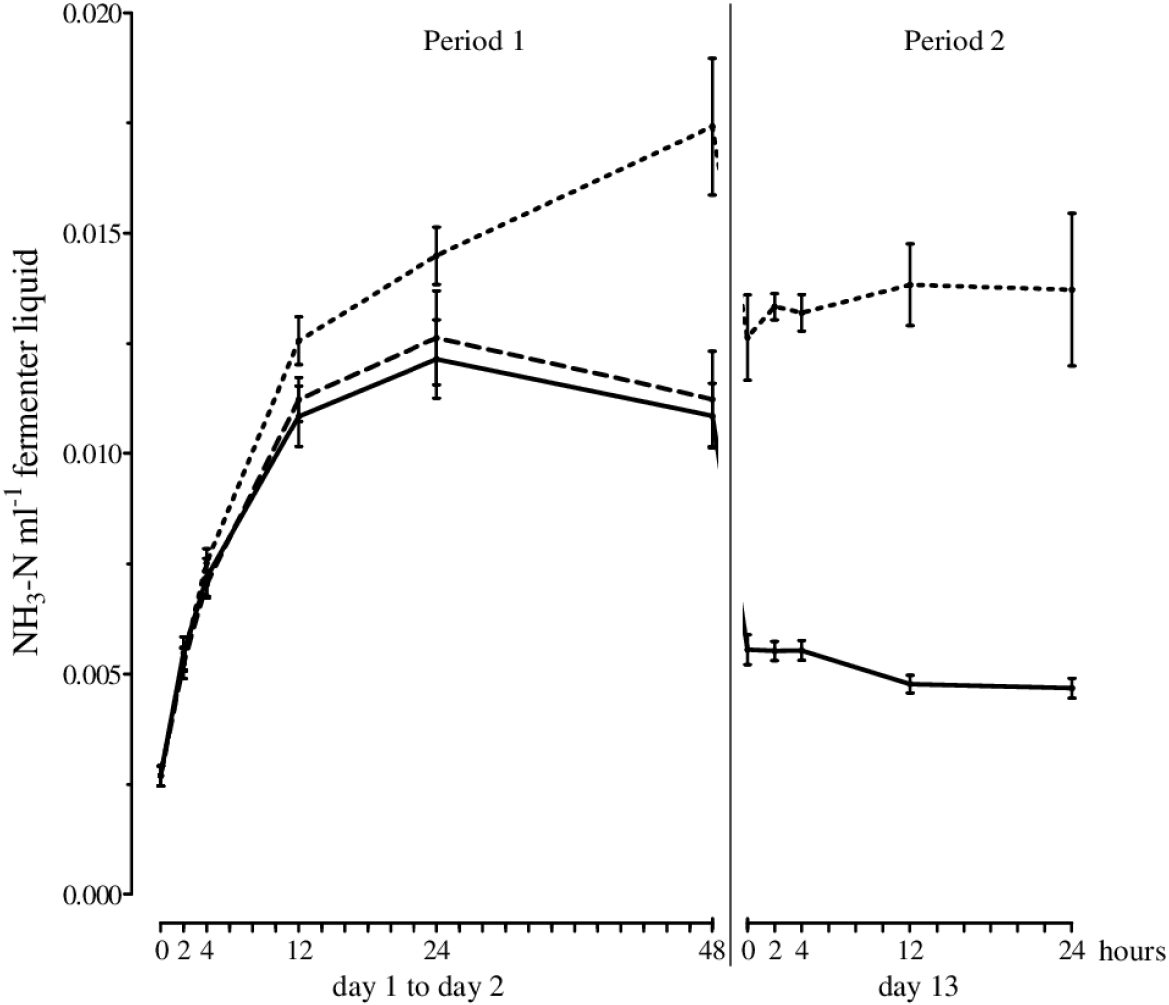
Ammonia-N (mmol in the fermenter liquid) over time for grass silage (dotted line), corn silage (solid line), and blank (dashed line; Mean, SEM; *n* ≥ 4). Significant differences between sampling times are described in the text.

The total gas production was not significantly affected by the silage used, but methane production was higher upon incubation of GS (Table 5). The degradation of organic matter and fiber fractions as well as the production of propionate and isobutyrate were also significantly higher for GS than for CS. Although the total SCFA, acetate, isovalerate, and valerate levels were not significantly affected by the silage used, the production of butyrate and the ratio of acetate to propionate were significantly higher for CS than for GS. CP degradation was similar between both silages but the amount of ammonia-N in the effluents and the efficiency of microbial CP synthesis were higher after incubation of GS. The ammonia-N in the fermenter liquids increased within the first 24 h of incubation irrespective of the forage source (Fig 7); however, after 48 h of incubation, the amount of ammonia-N was significantly higher for GS than for CS. The results from the blank controls were similar to those obtained for CS.

**Table 5:** Total gas and methane production, degradation of nutrients after 48 h of incubation, ammonia-N, and short-chain fatty acids (SCFA) in the effluent, and efficiency of microbial crude protein synthesis.

	CS^a^	GS^b^	*p*-value
pH	6.81 (0.04)	6.90 (0.03)	< 0.05
Total gas (ml day^-1^)	858 (51.2)	815 (57.8)	n.s.
Methane (ml day^-1^)	77.2 (4.27)	117 (13.7)	< 0.05
Degradation (%)			
Organic matter^c^	44.1 (0.94)	47.2 (0.97)	< 0.05
Crude protein^c^	67.4 (1.85)	66.5 (1.27)	n.s.
NDF^d^	7.86 (1.83)	17.7 (3.71)	< 0.05
ADF^e^	6.51 (2.17)	22.6 (1.10)	< 0.05
SCFA (mmol day^-1^)			
Total	34.2 (1.34)	31.9 (2.50)	n.s.
Acetate	13.6 (0.87)	12.6 (1.03)	n.s.
Propionate	5.08 (0.71)	6.63 (0.39)	< 0.05
Isobutyrate	0.32 (0.02)	0.55 (0.06)	< 0.05
Butyrate	8.79 (0.64)	6.00 (0.50)	< 0.05
Isovalerate	3.05 (0.32)	2.49 (0.55)	n.s.
Valerate	3.39 (0.26)	3.65 (0.34)	n.s.
Acetate:propionate	2.72 (0.44)	1.90 (0.06)	< 0.05
Ammonia-N in the effluent (mmol day^-1^)	3.04 (0.18)	7.41 (0.81)	< 0.05
Efficiency^f^	144 (2.70)	234 (5.37)	< 0.05

Data are expressed as the mean (SD); *n* ≥4,

^a^Corn silage,

^b^Grass silage,

^c^Corrected for contribution of solid associated microbes,

^d^Neutral detergent fiber without residual ash after α-amylase treatment,

^e^Acid detergent fiber,

^f^g microbial crude protein kg^-1^ degraded organic matter.

## Discussion

### Changes of the rumen microbial community composition during initial adaption to the Rusitec system

Among the microbial species investigated in the fermenter liquids and feed residues, the highest reduction over time was detected for protozoa, which occurred as early as 24–48 h following the start of incubation (Figs 1–6, Table 4). A considerable decrease of protozoa has also been described in previous *in vitro* studies [9, 10]. Compared to the generation intervals of protozoa [37], the turnover rate of the fermenter liquids in Rusitec systems is relatively high; thus, the protozoa might simply be washed out of the system [3, 38]. Protozoa are important H_2_ producers that play a key role in interspecies hydrogen transfer and methane production within the rumen microbial ecosystem [39]. Methanogens, conversely, are H_2_ consumers often showing also a physical association to protozoa [40]. However, in the present study no further decrease in the total number of methanogens was recognized in the fermenter liquids (Fig 1) after the first 24 h of incubation and no changes were found in the feed residues (Table 4), what might be expected following protozoa loss if the positive relationship between both groups is important in this system. One reason for this finding could be that in the absence of protozoa other rumen microbes that also produce H_2_ might increase their activity. No further decrease was found as well for the numbers of total bacteria after the first 24 h of incubation, indicating a change of the microbial community composition at the domain level during initial adaption to the Rusitec system. The changes observed for these three microbial groups in the fermenter liquids were similar to those found in the blank fermenters that contained only the solid phase from the rumen, which thus represents the approximate feed components of the diet of the donor animal. Hence, the observed shift within the microbial community cannot be solely linked to changes of the substrate provided for fermentation during *in vitro* incubation. Rather, the shift seemed to be a direct effect of the inoculum preparation and/or the Rusitec system itself that was still obvious after 13 days of incubation.

Changes in the microbial community composition during initial adaption to the *in vitro* system and to the forages were also seen on the species level. In fermenter liquids only the gene copy numbers of *R*. *albus* (Fig 2), *S*. *ruminantium*, *P*. *bryantii*, and *C*. *aminophilum* (Fig 3) were significantly higher after 48 h of incubation compared to those obtained at 24 h. The numbers of *C*. *aminophilum* increased to an even higher level than those of the inoculum, and for both *C*. *aminophilum* and *P*. *bryantii*, higher numbers were found in the feed residues compared to the solid phase (Fig 6).

On a domain level, the microbial community composition seemed to be similar at 48 h after the initiation of incubation and at the start of period 2. However, the differences in gene copy numbers found for *F*. *succinogenes* (Figs 2 and 5), *P*. *bryantii* (Figs 3 and 6), and *R*. *amylophilus* (Figs 3 and 5) in the fermenter liquids and feed residues, and for *C*. *aminophilum* (Figs 3 and 6) and protozoa (Fig 4) in the feed residues at days 2 and 13 indicated that adaption of these species to the *in vitro* system and forages was not completed by 48 h of incubation. We note that *R*. *amylophilus* (Figs 3 and 5) could not be quantified in period 2 because of the generation of non-specific PCR products that indicated either the presence of other *Ruminobacter* strains not amplified by the primers or a substantial decrease in the number of that species, what may indicate a lack of adaption to the Rusitec system.

### Diurnal changes of the microbial populations in the Rusitec system at the end of incubation (Period 2)

The provision of fresh substrate to the fermenters via a new feedbag at the beginning of period 2 led to increasing gene copy numbers of most microbial species in the fermenter liquids within the following 2 h of incubation (Figs 1–3). Belanche et al. [12] also detected the highest numbers of total bacteria 2 h after de novo incubation of a fresh bag with ryegrass or red clover in the Rusitec system. In the present study, only the protozoa and methanogens decreased in numbers in the fermenter liquids during the first 2 h after changing the feedbag. This postprandial decrease of protozoa in the fermenter liquids might be attributed to a migration from the fermenter liquids to new feed particles [41]. After breakdown of the available nutrients, subsequent migration back to the fermenter liquids might explain the high numbers of protozoa found 24 h after addition of the new substrate. This assumption is corroborated by the fact that in the feed residues, lower protozoa numbers were found after 48 h of incubation compared to those observed at 24 h (Fig 4). The abundance of methanogens in the fermenter liquids showed similar trends as for protozoa (Fig 1), in contrast to the situation observed following the initiation of the adaption period described previously, which might be caused by the fact that protozoa are important H_2_ producers [39] and that most of the ruminal methanogens use H_4_ and CO_2_ for methanogenesis [42].

In feed residues, the effect of sampling time was restricted to protozoa and *P*. *bryantii* (Figs 4 and 6). This is in accordance with Welkie et al. [43], who reported that the solid-associated microbial community showed less change in composition within and across feeding cycles compared to that seen for liquid-associated microbes using automated ribosomal intergenic spacer analysis (ARISA). Furthermore Craig et al. [44] reported that the level of particle-associated microbial organic matter was greatest soon after feeding. However, in the present study sampling of feed residues was only possible after 24 and 48 h of incubation; thus, possible changes in the populations of different particle-associated microbes directly after the initiation of incubation or after feeding could not be determined.

At 24 h after feed supplementation within period 2, the absolute numbers of almost all species examined were similar to those observed at the beginning of sampling on day 12, indicating that the microbial populations reached a dynamic steady state in the fermenter liquids within this *in vitro* system. In accordance with our results, Belanche et al. [12] identified similar microbial growth curves at days 10, 11, and 12 of incubation of ryegrass or red clover in the Rusitec system.

### Effects of the incubated forage source on the microbial populations and fermentation characteristics

The effect of forage source on various microbial species and the characteristics of fermentation are linked to differences in the chemical composition between silages. CS generally has a higher concentration of non-structural carbohydrates, primarily starch, and GS contains higher concentrations of CP (Table 1) and degradable fiber fractions (Table 5). Protozoa engulf and digest large numbers of bacteria and possess amylolytic as well as proteolytic [45] and cellulolytic activities [46]. This diversity in physiology might be the reason why no significant effect of forage source on the numbers of protozoa was detected in our study (Fig 4).

The forage source also did not affect the numbers of total bacteria or methanogens, which corresponds to the similar amounts of SCFAs and total gas production per day identified between the silages (Table 5). However, methane production was higher for GS compared to CS although the numbers of methanogens did not differ. One explanation for this observation might be underlying changes in methanogenic order composition, which would require that methanogenic orders with lower methanogenic activity were preferentially inhibited while those with higher methanogenic activities were enhanced [47]. It is not possible to test this hypothesis from results of the present study as detection was only performed on the group level.

Because of its cellulolytic activity, *F*. *succinogenes* was expected to be more abundant upon incubation of GS rather than CS. However, within period 2 we found higher numbers of this species in the fermenter liquids and in the feed residues after incubation of CS. Similar results have been reported by Lettat et al. [16], who found higher numbers of *F*. *succinogenes* in dairy cows fed with diets high in CS compared to those fed diets high in alfalfa silage. One explanation of this finding could be the differences in cell wall structure between C_3_ and C_4_ plants known to affect their degradation by microorganisms [48]. C_4_ plants as corn possess a much tougher cell wall compared to C_3_ plants from which GS is obtained. *F*. *succinogenes* is able to hydrolyze a wide variety of polysaccharides but can only utilize cellulose and its hydrolytic products for growth. Furthermore, the mechanism by which this species degrades cellulose is not completely understood but it is obvious that it stands in strong contrast to the strategies used by other cellulolytic microbes [49]. Thus, it could be possible, that *F*. *succinogenes* has an advantage in degrading C_4_ plant cell walls but this needs further research.

In the present study, the amount of fermented acid detergent fiber and CP were higher with fermentation of GS compared to that observed with CS. This is in accordance with the higher observed numbers of *R*. *albus* and *P*. *bryantii* because the former has cellulolytic activity and the latter ferments peptides and amino acids. The higher amount of ammonia-N in the effluent also confirms the assumption of higher amino acid fermentation when GS was incubated. *P*. *bryantii* is also involved in the degradation of hemicelluloses [50]; accordingly, a higher breakdown of structural carbohydrates such as hemicelluloses from GS was indicated by the higher neutral detergent fiber degradation and might in part be a result of the increased number of *P*. *bryantii*.

*R*. *amylophilus* requires starch or maltose as an energy source [50, 51]. Hence, higher numbers of this species found upon CS incubation compared to GS in period 1 were expected and our results are in accordance with those of Petri et al. [37], who found a higher relative abundance of *R*. *amylophilus* in cattle fed a diet with 49% compared to 35% starch. However, in period 2, the high supply of starch provided by CS did not lead to a further establishment of the *R*. *amylophilus* strains targeted by our primers, as previously discussed. Whether other strains were able to survive in the Rusitec system remains to be elucidated.

The ability of *S*. *ruminantium* to utilize starch [51], the products of starch hydrolysis [52], and the degradation products of cellulolytic bacteria [53] as energy sources is likely the reason for the missing effect of silage on that species.

*C*. *aminophilum* belongs to the class of hyper ammonia-producing bacteria (HAB). It uses only peptides and amino acids as energy sources while producing a high amount of ammonia [54]. Consequently, a higher abundance of *C*. *aminophilum* was expected to occur upon GS incubation. However, in the present study no differences in numbers of this organism were found between the silages. The higher amount of ammonia-N in the fermenter liquids (Fig 7) produced when GS was used could potentially not only be produced by this species but from others species involved in peptide and amino acid fermentation such as *P*. *bryantii*, which was shown to exhibit significantly increased numbers upon GS incubation.

Similar to the results of other *in vitro* studies [5, 19, 55] but contrary to the findings of Givens and Rulquin [56], we found that the efficiency of microbial CP synthesis was higher with incubation of GS compared to that observed with CS. This indicates that the content of available N as well as the N source could play important roles in the efficiency of microbial CP synthesis, as has been shown by several previous studies [4, 57].

The low acetate-to-propionate ratio in the current study seems to be specific for this *in vitro* system; similar ratios have been reported in previous studies [25, 58]. The low digestibility of fiber fractions might have resulted from lower cellulolytic activity due to the presence of corn starch negatively affecting microbial cellulolytic activity [18].

## Conclusion

To the best of our knowledge, this is the first study investigating the different ruminal microbial populations during initial adaption to a semi-continuous Rusitec system within the first 48 h of incubation. Our results suggest that on the domain level a stable microbial community composition was achieved after 48 h under the given incubation conditions. However, some species showed different numbers in period 2, indicating incomplete adaptation of these species to the *in vitro* system and forage after 48 h of incubation. Our findings on protozoa confirm the results of previous studies generated with other rumen models [3, 10] that showed a substantial initial decrease of the protozoa population *in vitro*. Consequently, we suggest that *in vitro* systems are only suitable to a limited extent to investigate the protozoan population.

In addition, our data suggested that the microbial populations reached a dynamic steady state in the fermenter liquids within this *in vitro* system after an adaption phase, and that this phase should last longer than 48 h for complete adaptation of all organisms. The different chemical composition of the two silages caused a different response of the microbial populations when each was used as the forage source. In particular, the growth of *F*. *succinogenes*, one of the most important cellulolytic bacteria in the rumen, was favored by the incubation of CS.
